# Interface-Limited Amperometric Cholesterol Biosensing in Ultrathin Pd-NPs-Based-Enzyme Films

**DOI:** 10.1021/acs.langmuir.6c01437

**Published:** 2026-07-10

**Authors:** Y.E. Silina, M. Koch, N. Korkmaz

**Affiliations:** † 9379 Saarland University, Department of Biochemistry, Campus B2.2, 66123, Saarbrücken, Germany; ‡ HTW saar − University of Applied Sciences, 66117, Saarbrücken, Germany; § KIST Europe − AI Convergence Cluster, 375133 Korea Institute of Science and Technology, 66123, Saarbrücken, Germany

## Abstract

This manuscript investigates the limitations of enzyme–electrocatalyst coupling in one-step designed amperometric cholesterol biosensors. One-step electrodeposition from Pd-ChOx-Nafion electrolyte produces a bioinorganic hybrid sensing layer, while preserving the enzyme’s native conformation and biocatalytic activity. Despite structural coupling between Pd and ChOx, the amperometric response remains dominated by enzymatic H_2_O_2_ production, indicating that direct electronic communication between ChOx and the electrode is not established. More specifically, we show that direct electron transfer (DET) cannot occur even in ultrathin electrodeposited nanostructured films, likely due to oxygen-dominated electrochemistry. Furthermore, the sensitivity of cholesterol determination using Pd-NPs/ChOx/Nafion-modified electrodes was independent of layer thickness and architecture, highlighting the limitations of the Pd-NPs–enzyme interface. These findings provide mechanistic insight into ultrathin enzyme–electrocatalyst interfaces and are expected to impact the development of next-generation cholesterol biosensors.

## Graphical abstract

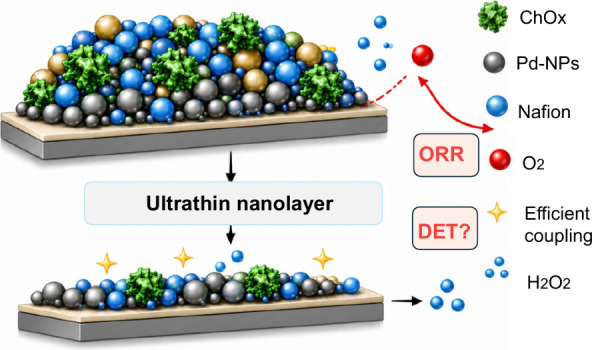

## 1 Introduction

The development of amperometric biosensors based on cholesterol oxidase (ChOx) for cholesterol determination is one of the most challenging tasks in the field of enzymatic amperometric biosensing. This is due to several factors, including the extremely low solubility of cholesterol in water, the poor immobilization capability of cholesterol oxidase, possible changes in its conformational structure on the electrode surface, and a decrease in its activity in a surfactant-containing environment (e.g., Triton, Tween, or PEG) used to dissolve cholesterol. In addition, the enzymatic behavior of the resulting biosensors often shows poor agreement with the Michaelis–Menten model.^1–3^

Furthermore, in contrast to other physiologically important metabolites (e.g., glucose, uric acid, and dopamine), cholesterol itself is not electroactive. Therefore, amperometric cholesterol biosensors rely exclusively on a single signal read-out strategy based on the detection of hydrogen peroxide produced during the enzymatic reaction.^4,5^ Hydrogen peroxide (H_2_O_2_) has a high oxidation potential; therefore, the use of mediators (such as Prussian blue, ferrocene, or Os polymers) or electrocatalysts (*viz*., noble metals, Pt-NPs, Pd-NPs) is essential.^6–8^ The selected peroxide-sensitive mediator or electrocatalyst must not only provide a high sensitivity and a low oxidation potential for H_2_O_2_ to avoid interference from other compounds present in real samples but also exhibit fast kinetics toward its decomposition. Otherwise, an enzyme rapidly degrades in the presence of its own byproduct. In addition, the use of surfactants that are not compatible with enzymatic activity, although necessary to increase cholesterol solubility, leads to significant matrix effects that mask the H_2_O_2_-related signal and cause rapid and irreversible deactivation of the enzyme.^9–11^

To address the aforementioned limitations, one-step (OS) electrodeposition from multiple electrolyte solutions containing oxidases and Pd precursors, resulting in the formation of a hybrid biosensing layer with an environmentally friendly interface, could be an effective alternative.^12,13^ This methodology enables the fabrication of thin hybrid layers composed of Pd nanoparticles (Pd-NPs) with enzymes/oxidases immobilized within the same layer. The resulting thin functional biosensing layer facilitates rapid H_2_O_2_ decomposition, thereby preserving enzyme activity. Moreover, in such thin layers, all enzyme molecules are readily accessible to the electrode surface, supporting fast mass transport. These structural features may also facilitate interfacial electron exchange and potentially enable direct electron transfer (DET) at enzyme–electrode interfaces.

DET in enzymatic biosensors has been widely demonstrated on nanostructured noble metal electrodes (Au, Pt), where NPs-modified thin films provide a reduced electron-transfer distance and improved enzyme–electrode coupling (with oxidases often used as model systems), thereby enabling efficient interfacial electron exchange.^14,15^ By reducing the electron-transfer distance and improving enzyme–electrode coupling through tailored immobilization strategies, such hybrid architectures can increase current density, sensitivity, and overall analytical performance. Importantly, the interplay between enzyme structure, prosthetic redox centers, and nanostructured electrode design is a key factor governing whether efficient DET can be achieved, making rational engineering of both components essential for the development of high-performance third-generation biosensors.^16,17^

Herein, the functional cholesterol-sensitive ultrathin layer was formed via OS electrodeposition (ED) performed from multiple electrolytes containing Pd precursors, FAD-dependent enzyme cholesterol oxidase (ChOx), and Nafion (Naf) used as a binding agent. During the course of the investigations, it was revealed that although ChOx was electrodeposited in its biochemically active form, a Pd-NPs electrocatalyst was simultaneously formed and showed high activity toward H_2_O_2_ decomposition, yet the resulting biosensor still exhibited certain performance limitations. More significantly, under oxygen-containing conditions, the ultrathin configuration and increased enzyme loading did not lead to a measurable signal enhancement or DET contribution on Pd-NPs-based electrodes. Instead, the efficiency of interfacial electron communication was primarily determined by the quality of the surface contact/coupling between the enzyme (ChOx) and the electrocatalyst (Pd-NPs), rather than by film thickness, electrode design, or enzyme concentration.

These findings provide mechanistic insights into the persistent dominance of oxygen reduction over DET in the hybrid ultrathin layer, highlighting limitations in Pd-NPs–ChOx interface coupling, and are expected to guide the further design of cholesterol-sensitive amperometric biosensors.

## 2 Experimental Section

### 2.1 Chemicals and Materials

H_2_PdCl_4_, (NH_4_)_2_HPO_4_, Na_2_HPO_4_·12H_2_O, 25% NH_4_OH, Nafion117 (Naf) solution (5% *v/v*), cholesterol (≥99%), cholesterol oxidase, ChOx, lyophilized powder, ≥ 50 units/mg protein, recombinant, expressed in *E. coli*, d-glucose, flavin adenine dinucleotide disodium salt hydrate (FAD, ≥ 95% (HPLC), ethanol (EtOH), iso-propanol, Triton X-100, hydrogen peroxide (H_2_O_2_, 30% (*w/w*) in H_2_O) were received from Merck (Darmstadt, Germany). Oxygen-free OXCAL buffer (oxygen content 0 μM/L) was obtained from (PyroScience GmbH, Aachen, Germany).

DRP-110DGPHOX screen-printed electrodes (SPEs), modified with graphene oxide (GO), were obtained from DropSens (Metrohm, Germany).

pH measurements were conducted using a HORIBA LAQUATWIN PH-22 pH sensor (MMM Tech Support GmbH and Co. KG, Germany).

### 2.2 Substrate Preparation

Cholesterol stock solutions were prepared in a mixture of phosphate buffer (pH 7.0 ± 0.2), 4% Triton X-100, and 10% iso-propanol (hereafter referred to as mixture A), following a previously optimized protocol.^1,2^

### 2.3 Fabrication of OS Cholesterol-Sensitive Electrodes

Electrodes modified with a one-step (OS) electrodeposited (ED) layer were fabricated at room temperature (20 ± 2 °C) using the following protocol: Pd-electrolyte (pH 9.3 ± 0.2) was premixed with 2% Nafion, Naf (stock solution diluted with EtOH, adjusted to pH 8.3 ± 0.1), and 3 mg/mL ChOx solutions (pH 7.0 ± 0.2) in a 1:1:1 (*v/v/*v) ratio, resulting in 1 mg/mL ChOx in the mixture. Ten μL of the mixture was placed on SPEs/GO, followed by electrodeposition at −2.5 mA for 30 s,^18^ resulting in the formation of the hybrid Pd-NPs/ChOx/Naf biosensing layer.

The inclusion of Naf in the sensing layer was essential for preserving enzyme activity and stabilizing Pd-NPs within the hybrid matrix. In this configuration, Naf does not act as a permselective membrane but as a structural component embedded in the Pd-based layer, which prevents its elution upon exposure to iso-propanol (mixture A, section 2.2) used for cholesterol stock preparation. After preparation, the electrodes modified with Pd-NPs/ChOx/Naf layers were carefully washed with DI water and stored at +4 °C prior to electrochemical studies.

### 2.4 Electrochemical Studies

The fabricated electrodes modified with Pd-NPs/ChOx/Naf were characterized by cyclic voltammetry (CV) in a potential window of – 0.4 to 0.4 V using a one-channel PalmSens4 potentiostat (PalmSens, Utrecht, The Netherlands) at a scan rate of 50 mV/s, unless otherwise specified, in background electrolyte (phosphate buffer or mixture A), cholesterol solutions, and hydrogen peroxide samples.

Electrode specificity was evaluated in continuous amperometric mode (AM) at an applied potential of 0.2 V using blank solution (mixture A), H_2_O_2_, glucose, and cholesterol solutions prepared in mixture A.

Cholesterol quantification was performed in the hydrogen peroxide (H_2_O_2_) detection mode according to a previously optimized multistep amperometric (MAM) protocol.^19^ Briefly, a first polarization at −0.2 V was applied for 60 s to reduce palladium oxides on the surface of the hybrid layer (level 1), followed by H_2_O_2_ signal acquisition at 0.2 V for 30 s (level 2).

The electroactive surface area (ECSA) of the Pd-NPs-based hybrid layer was determined using a previously established protocol for Pd-NPs-modified electrodes.^20,21^ The peak area of the cathodic peak recorded in phosphate buffer at pH 7 was integrated to calculate the charge passed (μC) relative to the mass of the electrodeposited Pd. The amount of ED Pd-NPs (inorganic component/electrocatalyst) deposited at room temperature within the entire biosensing layer (Pd-NPs/ChOx/Naf) was determined in chronopotentiometric (CP) mode using a 1 M HCl droplet at a constant current of 0.1 mA for 200 s.^20,21^

The deposited mass of Pd-NPs in the Pd-NPs/ChOx/Naf layer was found to be 2.75 ± 0.35 μg, and the ECSA was determined to be 0.40 ± 0.14 cm^2^, resulting in a SECA (ECSA/mass) of 14.6 ± 5.4 m^2^/g.

### 2.5 Oxygen Mini-Sensor Studies and Determination of ChOx Activity

The biochemical transformation of cholesterol catalyzed by ChOx is accompanied by oxygen consumption:^22^

1 $$\mathrm{Cholesterol}+O_{2}\to \mathrm{cholestenone}+H_{2}O_{2}$$

Therefore, ChOx activity, either in its native form or after mixing with the Pd-electrolyte and Naf, can be conveniently assessed by monitoring oxygen consumption during its reaction with cholesterol.

For this purpose, an OXR430 fiber-optic oxygen minisensor (PyroScience GmbH, Aachen, Germany) was inserted into a 150 μL droplet of cholesterol solution placed on a Petri dish, followed by the addition of 10 μL aqueous ChOx with a stock concentration of 3 mg/mL, resulting in a final ChOx concentration of 0.19 mg/mL (total volume 160 μL).

Remarkably, agreement with the Michaelis–Menten model^23–25^ for a droplet containing cholesterol and ChOx was observed only in the concentration range of 0–100 μM (SI, Figure S1A, *curves a–d*). At cholesterol concentrations above 100 μM (SI, Figure S1A, *curves e,f*), oxygen consumption was almost negligible, indicating enzyme saturation: oxygen is either consumed extremely rapidly or substrate-induced enzyme inhibition occurs. Thus, if the *Y*-axis is interpreted not as reaction rate but as oxygen concentration, the entire curve exhibits an S-shaped profile, confirming rapid enzyme saturation, SI, Figure S1B.

This behavior is consistent with the expectation that increasing substrate concentration initially enhances the reaction rate, leading to an increased enzymatic oxygen consumption and a concomitant decrease in dissolved oxygen that strongly suggests a regime change above ∼ 100 μM cholesterol: at concentrations of 1–3 mM, oxygen consumption rapidly collapses to zero, indicating a departure from classical Michaelis–Menten kinetics. Under these conditions, oxygen depletion and/or substrate-induced inhibition of cholesterol oxidase dominate the system kinetics.

### 2.6 Atomic Force Microscopy (AFM) Studies and Determination of the Thickness of the OS Hybrid Layer

The surface morphology and thickness of the electrodeposited layer were characterized using AFM. Measurements were performed with a PARK NX 10 AFM system (Park Systems, South Korea) in noncontact mode under ambient laboratory conditions. AFM ACTA (NCHR) probes compatible with the Park NX 10 system were obtained from Schaefer Technologie GmbH (Germany).

Topographical images were captured over a scan area of 1 μm × 0.74 μm with a resolution of 512 × 380 pixels. 3D topography image was constructed using the XEI image processing software (Park Systems, South Korea). The thickness of the hybrid biosensing layer deposited on SPEs/GO was determined as follows: one height profile was extracted as shown on the AFM topography image. Eighteen peaks were selected and the corresponding heights were estimated using the profile extraction tool of Gwyddion software. Average thickness was calculated as mean ± standard deviation.

### 2.7 TEM Analysis

To confirm the codeposition of Pd-NPs together with ChOx and Naf within a single hybrid layer, TEM investigations were performed on modified SPEs/GO. Small fragments of the functional biosensing layer were obtained by scratching the surface with P180 sandpaper. The resulting material was dispersed by placing an ethanol droplet onto a holey carbon grid (Plano, Wetzlar, type S147–4). Bright-field TEM imaging was carried out at an accelerating voltage of 200 kV using a JEOL JEM 2100 Lab6 microscope (Akishima, Tokyo, Japan) equipped with an HR pole piece and a Gatan Orius SC1000 CCD camera (Pleasanton, CA, USA).

### 2.8 Quartz Crystal Microbalance (QCM) Studies

The mass of the hybrid Pd-NPs/ChOx/Naf layers deposited on the electrodes at – 2.5 mA for 30 s was determined using a QCM200 system (SRS, Scientific Instruments GmbH) with gold-coated quartz crystals (5 MHz resonance frequency). Briefly, hybrid layers were electrodeposited from the same multiple electrolyte used for SPEs/GO modification (section 2.3) under identical electrodeposition parameters. Mass of deposits were calculated using the *Sauerbrey* equation:^26^

2 $$\Delta m=\frac{\Delta F}{C}\cdot A$$

where ΔF – is the frequency shift, Hz; C – is the mass sensitivity constant (for 5 MHz AT-cut quartz, C ≈ 17.7 Hz·cm^2^/ng; A – is the electrode area (0.4 cm^2^).

Knowing the mass from equation ([Disp-formula eq2]), the thickness of the deposited biosensing layer can also be found:

3 $$d=\frac{m}{\rho \cdot A}$$

where *d* – thickness of the film, *A* – is the electrode area (0.4 cm^2^), ρ – density of the film material (the density was taken for the hybrid material). For the electrolyte solution, equal parts (*v/v/v*) of palladium precursor electrolyte (ρ_1_), Naf (2%) (ρ_2_), and ChOx (ρ_3_) were used. The average density of the formed layer was taken as

4 $$\rho =\frac{\rho 1+\rho 2+\rho 3}{3}=\frac{12.02+2.0+1.3}{3}=5.11g/{\mathrm{cm}}^{3}$$

## 3 Results and Discussion

### 3.1 Characterization of One-Step (OS)-Designed Pd-NPs/ChOx/Naf-Based Electrode

First, the OS fabricated electrode was characterized using TEM and AFM. TEM images showed a homogeneous, thin, and continuous organic matrix (ChOx/Naf) with incorporated Pd-NPs with a size of 20–30 nm (SI, Figure S2A), while AFM analysis indicated a hybrid layer thickness of 18.4 ± 9.7 nm (SI, Figure S2B-D).

The thickness (d) of the hybrid layer, calculated from QCM studies (Figure 1) using the same concentrations of ChOx, Naf, and Pd-electrolyte, was consistent with the nanometer-scale thickness measured by AFM. Minor differences in thickness between the two techniques can be attributed to the substrates used: SPEs/GO for AFM versus gold crystals for QCM studies (see the Experimental Section).

**Figure 1:**
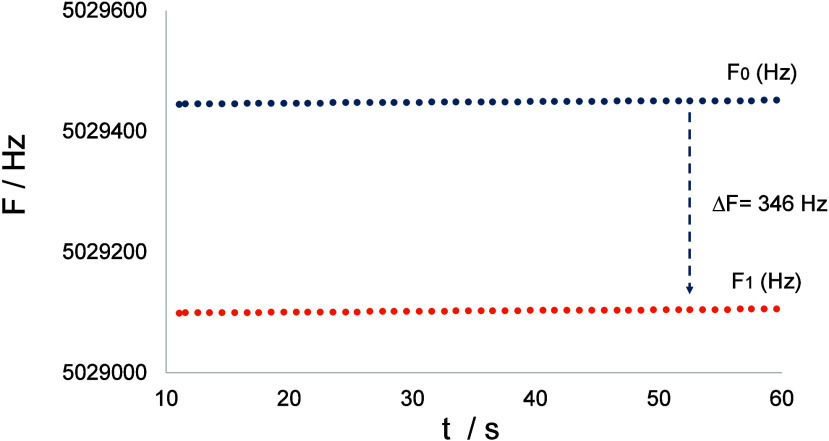
QCM frequency response curves of the gold QCM sensor (F_0_) before and after electrodeposition of the Pd-NPs/ChOx/Naf layer (F_1_). The mass of the deposited layer was estimated using the *Sauerbrey* equation as Δm ≈ 6.12 μg/cm^2^, corresponding to a total mass on the electrode area (0.4 cm^2^) of *m*_total_ ≈ 2.45 μg. The layer thickness (d), calculated from the hybrid layer density (5.11 g/cm^3^), is ∼ 12.0 nm.

The formation of such an ultrathin hybrid biosensing nanolayer is expected to facilitate improved electron transfer within the film.^27^ Moreover, the ultrathin thickness of the layer may promote possible DET by bringing the FAD redox center closer to the electrode surface.^28–30^

Next, it was important to define the electrochemical rate-limiting process occurring in this hybrid biosensing layer. The electrode, modified with a Pd-NPs/ChOx/Naf layer, was tested at various scan rates in conventional phosphate buffer, both in the presence and absence of oxygen (using OXCAL buffer), while maintaining the same pH.

Briefly, the anodic (I_pa_) and cathodic (I_pc_) peak currents recorded from the Pd-NPs/ChOx/Naf-modified electrode increased linearly with the scan rate over the range of 5–500 mV/s, with correlation coefficients greater than 0.97 (Figure 2A,B).

**Figure 2:**
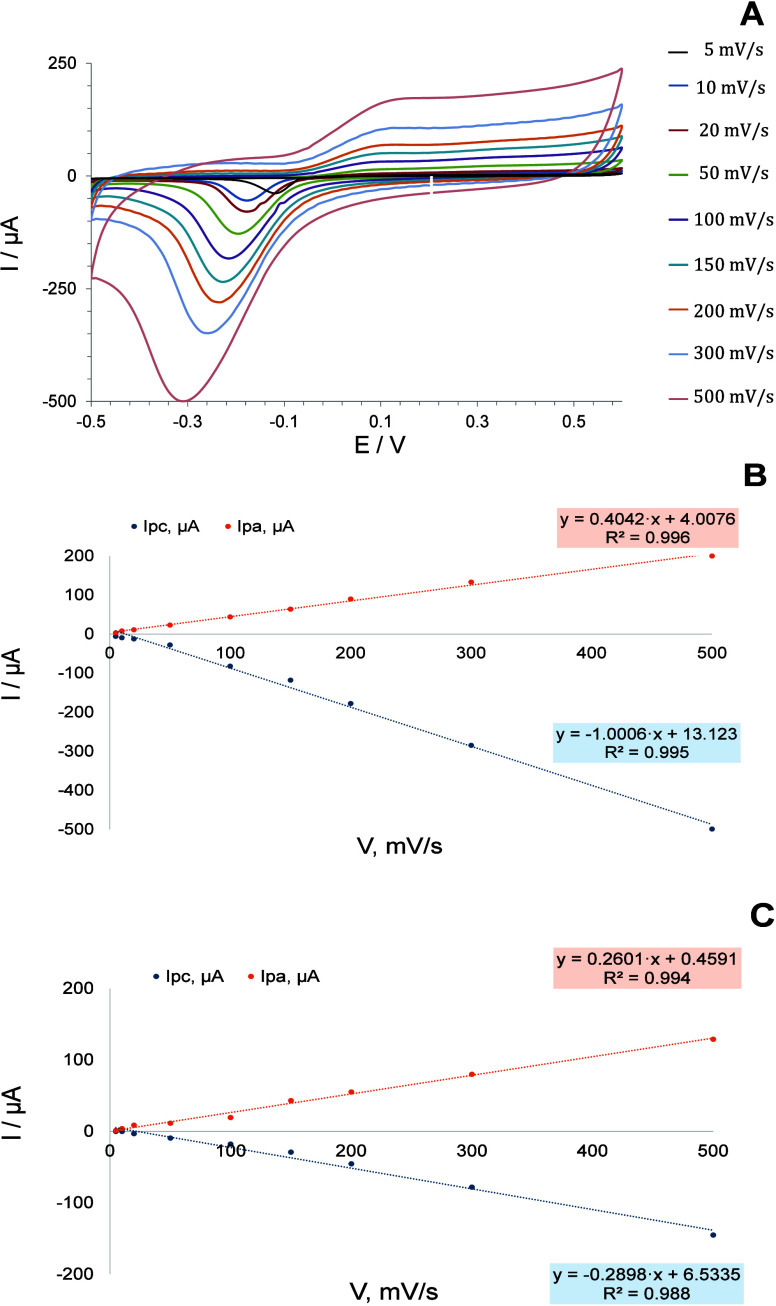
(**A**) Cyclic voltammograms, CV, (2D scans shown) obtained from OS designed Pd-NPs/ChOx/Naf-modified electrode in phosphate buffer (pH 7.0 ± 0.2) at various scan rates measured from 5 to 300 mV s^–1^ (from inner to outer profiles), respectively, and (**B**) plots of peak currents vs. the scan rates (vs. Ag/AgCl) at the ambient conditions (**B**) and in the absence of oxygen, OXCAL buffer (**C**). *Note*: in (**B,C**) I_pe_ – anodic peak current; I_pc_ – cathodic peak current.

This linear dependence suggests that the redox process is surface-controlled, indicating an adsorption-dominated electron transfer in the hybrid biosensing layer.^31^ Notably, the higher slope observed for the cathodic peak current compared to the anodic peak suggests asymmetric charge-transfer kinetics, with the reduction process being more favorable at the Pd-NPs/ChOx/Naf-modified electrode (Figure 2B, I_pc ≫_ I_pa_).

The lower slope (I_pa_) observed for the anodic peak current can be attributed to the formation of PdO on the surface of the Pd-NPs during the positive potential sweep, which partially passivates the electrode. In contrast, the cathodic process involves the rapid reduction of surface PdO back to metallic Pd (Pd^0^), resulting in a significantly higher cathodic current and slope (I_pc_, Figure 2B).

At the same time, scanning the same electrode modified with the same biosensing layer in the absence of oxygen did not reveal any asymmetry between I_pa_ and I_pc_ (Figure 2C, I_pa ≈_ I_pc_), indicating a pronounced background contribution of ORR to the cathodic current in the presence of oxygen (Figure 2B, I_pc ≫_ I_pa_). Notably, in this case ORR not only adds current but also distorts the sensor’s electroanalytical function.

### 3.2 Electroanalytical Performance of OS Designed Pd-NPs/ChOx/Naf-Modified Electrode in Cholesterol Solutions: Electrocatalyst or Enzyme Limitations?

#### 3.2.1 Electrochemical Characterization and H_2_O_2_ Sensing Performance

The CV plots recorded from the Pd-NPs/ChOx/Nafion-modified electrode in surfactant mixture A exhibited a characteristic cathodic peak at approximately – 0.3 V (Figure 3A, *plot a*), corresponding to the electroreduction of palladium oxides and ORR on the Pd-NPs electrocatalyst surface.^19,20^

**Figure 3:**
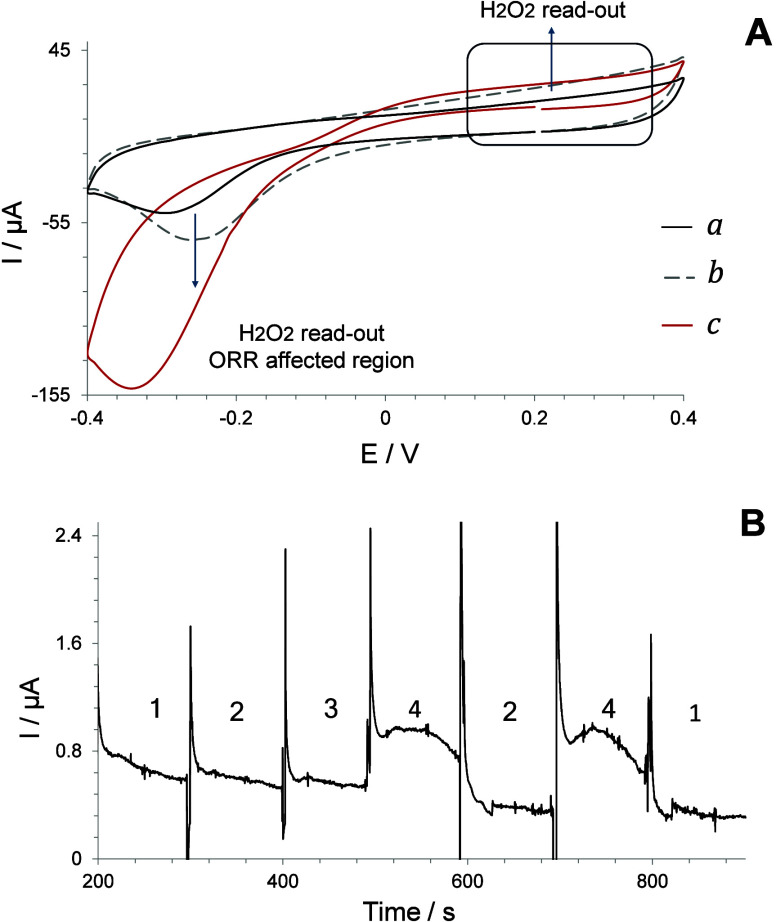
(**A**) CV plots (2D scans shown) recorded from OS ED Pd-NPs/ChOx/Naf-modified electrode at 50 mV/s in a droplet of: *a* – surfactants mixture (mixture A); *b* – 3 mM cholesterol in mixture A; *c* – 1 mM H_2_O_2_ in mixture A; *Note*: signal read-out region is marked by a rectangle. (**B**) Representative specificity test recorded at 0.2 V from the electrode modified with OS ED Pd-NPs/ChOx/Naf layer: 1 – mixture A (matrix/surfactant environment); 2 – 2 mM glucose in mixture A; 3 – 100 μM cholesterol in mixture A; 4 – 2 mM cholesterol in mixture A. The experiment shown in (**B**) was performed in triplicate with reproducible results.

More significantly, exploring the electrode in CV mode in cholesterol-containing solutions showed that the enzymatic activity of ChOx was preserved after immobilization with Pd-NPs and Naf via electrodeposition (ED). Briefly, two distinct signals were observed on the Pd-NPs/ChOx/Naf-modified electrode in the presence of cholesterol: a cathodic peak at – 0.25 V corresponding to an additive signal arising from electroreduction of palladium oxides (i), ORR (ii), and H_2_O_2_ (iii) and an anodic wave at 0.2–0.4 V, corresponding exclusively to the Pd-NPs-catalyzed oxidation of H_2_O_2_, which is generated *in situ* as a byproduct (Figure 3A, see for comparison CV *plots a, b*). These data suggest that structural coupling between Pd-NPs and ChOx occurred during ED, preserving the biochemical activity of ChOx for cholesterol oxidation and electrochemical activity of Pd-NPs toward H_2_O_2_ electrooxidation.

Considering the strong catalytic activity of Pd-NPs toward ORR at neutral pH, cathodic detection of H_2_O_2_ is prone to significant interference^12^ (see also Section 3.1). Therefore, to minimize the impact of oxygen, cholesterol analysis, based on H_2_O_2_ detection as a byproduct by Pd-NPs/ChOx/Naf-modified electrodes, should be carried out in the anodic range of potentials. Notably, this interference is not specific to Pd-NPs but is a general feature of noble-metal nanostructures (e.g., Pt and Au) exhibiting ORR activity within the same cathodic potential window.

The addition of H_2_O_2_ to the same surfactant-containing matrix A (spiked sample) confirmed that the recorded signal indeed corresponds to H_2_O_2_ generated by the enzymatic reaction (Figure 3A, CV *plot c*). However, it should be noted that the anodic current generated in the presence of 1 mM H_2_O_2_ spiked into the same matrix was significantly higher than the current recorded from the same electrode even in the presence of the higher cholesterol concentration (Figure 3A, shown for 1 mM H_2_O_2_ and 3 mM cholesterol, see in comparison CV *plots b and c*). This observation indicates that Pd sites remain available in the hybrid biosensing layer for effective interactions with free hydrogen peroxide on one hand and that the electrochemical signal is limited by the rate and efficiency of enzymatic H_2_O_2_ generation (biochemical step) on the other hand.

Meanwhile, the specificity of the biochemical reaction, governed by the type of immobilized enzyme (ChOx) within the design of OS designed Pd-NPs/ChOx/Naf-modified electrode, and the proper electrochemical signal read-out (H_2_O_2_ oxidation catalyzed by Pd-NPs), were also confirmed in AM mode using a static applied potential of 0.2 V, Figure 3B. It can be observed that the amperometric signal of the Pd-NPs/ChOx/Naf-modified electrode returns to the baseline after sequential additions of cholesterol, demonstrating the reversibility and reproducibility of the sensor response.

Notably, the instantaneous saturation of ChOx observed at cholesterol concentrations of 1–3 mM in solution (SI, Figure S1) was not detected after enzyme immobilization on the electrode surface. Instead, a linear dependency was observed over the investigated cholesterol concentration range (approved during cholesterol quantification in MAM mode, see Experimental section, Figure 4A). The sensitivity achieved was in the range of 1.0 – 1.1 μA·μM^–1^·cm^–2^, which is in line with values reported in the literature for hydrogen peroxide-dependent read-out using nanoparticles.^32–34^

**Figure 4:**
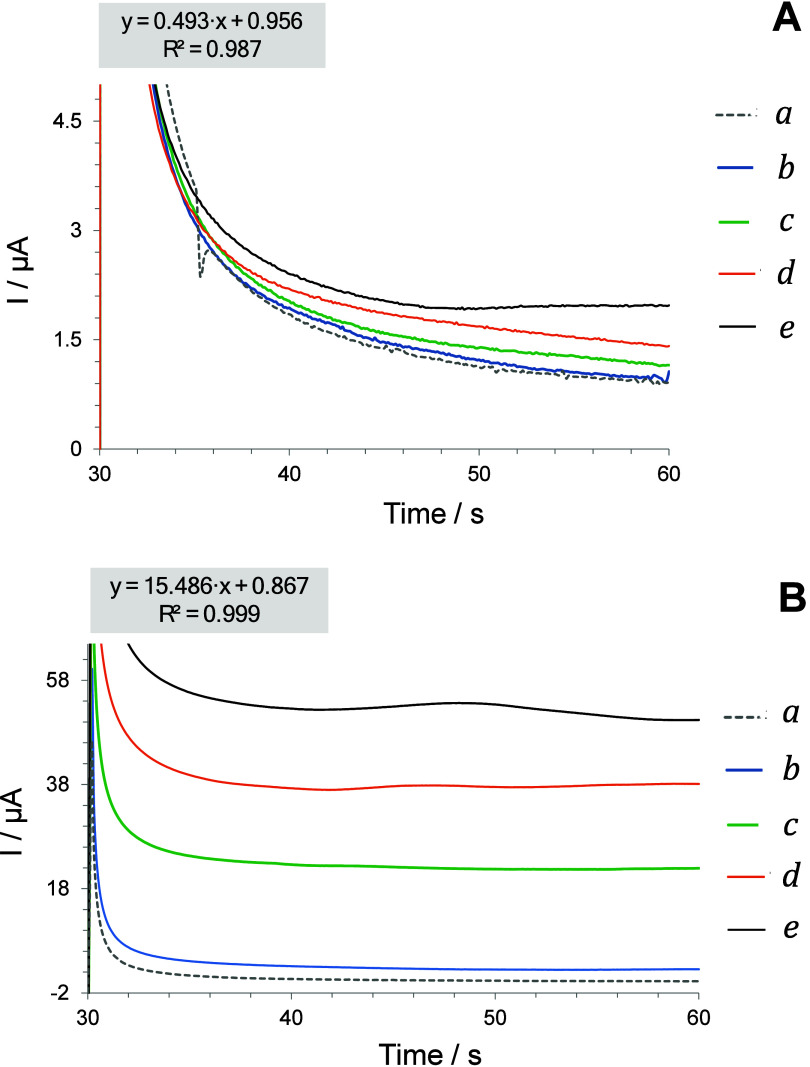
(**A**) Representative current responses recorded in MAM mode from the OS ED Pd-NPs/ChOx/Naf-modified electrode at 0.2 V in a droplet of: (*a*) mixture A (0 μM cholesterol); (*b*) 100 μM cholesterol; (*c*) 1000 μM cholesterol; (*d*) 2000 μM cholesterol; (*e*) 3000 μM cholesterol. (**B**) Representative current responses recorded in MAM mode from the same electrode in the presence of H_2_O_2_: (*a*) 0 μM (mixture A); (*b*) 100 μM H_2_O_2_; (c) 1000 μM H_2_O_2_; (*d*) 2000 μM H_2_O_2_; (*e*) 3000 μM H_2_O_2_. *Note*: The pH of all solutions prepared in mixture A was set at 7.02 ± 0.2. The measurements were repeated in triplicate using the same electrode, yielding reproducible responses.

More significantly, the above-mentioned mechanistic interpretation regarding the minimal influence of the electrocatalyst (Pd-NPs) on the overall electrode response was also verified in MAM mode through the quantification of both analytes (cholesterol and hydrogen peroxide). Thus, the sensitivity to H_2_O_2_ formed as a byproduct of the interaction between ChOx and cholesterol in the same matrix/mixture A (y = 0.4936·x + 0.9563, LDR = 100 μM – 3 mM) was several orders of magnitude lower compared to externally added H_2_O_2_ measured at the same electrode with the same sensing layer design and Pd-NPs (y = 15.486·x + 0.8667, providing linear detection range, LDR, 100 μM – 3 mM), Figure 4B.

The comparison of calibration slopes indicates that the amperometric response to H_2_O_2_ generated enzymatically by ChOx in the presence of cholesterol (∼0.493 μA/μM) is only ∼ 3% of that observed for externally added H_2_O_2_ (∼15.486 μA/μM), highlighting an interface-limited mechanism. At the same time, it confirms that Pd-NPs within the same biosensing layer efficiently detect H_2_O_2_ and do not limit the electrode performance. Accordingly, the overall electrode response is likely weakly influenced by the properties of the inorganic component/electrocatalyst (Pd-NPs), such as their ECSA or surface density.

This assumption was subsequently confirmed by a model experiment using individual Pd-NPs deposited on SPEs/GO from an electrolyte with the same concentration of Pd precursors as in the multiple electrolyte. Briefly, by maintaining the cathodic current at a constant level (−2.5 mA) and varying the deposition time from 30 to 90 s, it was possible to increase the mass of deposited Pd-NPs from 1.93 μg to 4.14 μg (SI, Figure S3), while the ECSA decreased from ∼ 0.35 to 0.19 cm^2^ and the specific SECA decreased from ∼ 14.5 to 4.66 m^2^/g. At the same time, changes in the mass (loading of Pd-NPs) and variations in SECA of Pd deposits had almost no effect on the sensitivity of H_2_O_2_ determination (Table 1).

**Table 1:** Electrochemical Characterization and Electroanalytical Performance toward H_2_O_2_ of Electrodes Modified by Individual Pd-NPs Produced at Varied Deposition Times and Evaluated in CV Mode at 50 mV/s^a^

Deposition mode of Pd-NPs	Calibration formula^b^	R^2^	ECSA (cm^2^)	Mass, μg	SECA, m^2^/g	*S* ^c^, μA·mM^–1^·cm^–2^
–2.5 mA, 30 s	y = 15.16·x – 1.1315	0.975	0.35 ± 0.081	1.93	14.58	52.31
–2.5 mA, 60 s	y = 12.32·x – 0.5785	0.987	0.21 ± 0.004	3.03	6.36	58.66
–2.5 mA, 90 s	y = 10.44·x – 0.1555	0.995	0.19 ± 0.002	4.14	4.66	54.09

a Calibrations with H_2_O_2_ were performed in *mixture A* at pH 7 ± 0.02 in MAM mode and signal read-out at 0.2 V;

b evaluated in he LDR = 100 μM – 3 mM;

c sensitivity of H_2_O_2_ determination by Pd-NP-modified electrodes.

To summarize, the observed insufficient currents corresponding to the oxidation of enzymatically formed H_2_O_2_ at the Pd-NPs/ChOx/Naf electrode cannot be explained by a nonoptimal electrocatalyst (Pd-NPs) design within the hybrid biosensing layer but can instead be attributed to the low enzyme loading. Therefore, the electrochemically addressable flux of H_2_O_2_ within the hybrid sensing layer is significantly lower than that theoretically possible.

#### 3.2.2 Impact of Enzyme Loading in OS ED Pd-NPs/ChOx/Naf-Modified Electrode

To assess the impact of the enzyme loading on the enzymatically generated H_2_O_2_ signal in the ultrathin hybrid biosensing layer, the concentration of ChOx in the electrolyte solution was increased from 1 mg/mL to 4 mg/mL. Meanwhile, the amounts of Pd precursors and Nafion were kept constant.

Notably, according to oxygen mini-sensor measurements performed in multiple electrolyte solutions, the linear increase in ChOx concentration was accompanied by a proportional increase in biochemical activity toward cholesterol decomposition in solution (SI, Figure S4). These results indicate that ChOx preserves its biochemical activity in multiple electrolytes and that its activity increases proportionally with increasing enzyme concentration. In other words, this experiment demonstrates the absence of local enzyme inactivation, at least in multiple electrolyte-containing Pd precursors and Naf.

After electrodeposition from multiple electrolyte solutions containing 1, 2, and 4 mg/mL of ChOx, the electroanalytical response (sensitivity) of the formed electrodes was compared as a function of the thickness of OS ED hybrid layers (Figure 5). The thickness of the formed hybrid biosensing layers was evaluated using a QCM system with QCM Au-quartz crystals following the described ED procedure used for SPEs/GO. The increase in ChOx concentration from 1 mg/mL to 4 mg/mL resulted in a linear increase in the layer thickness.

**Figure 5:**
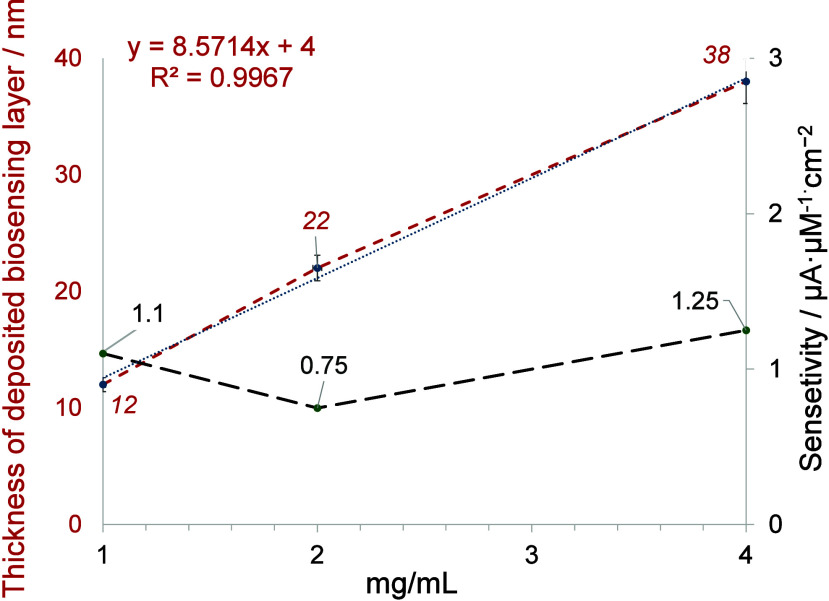
Overlay of the dependence between the thickness of the deposited hybrid layers (nm), evaluated by QCM, and the sensitivity of cholesterol detection using SPEs/GO modified with the same hybrid Pd-NPs/ChOx/Naf layers, as a function of ChOx concentration (mg/mL) in the multiple electrolyte solution.

At the same time, the nearly constant sensitivity of the electrodes toward cholesterol detection, regardless of the enzyme concentration loaded in the multiple electrolyte or the codeposited thickness of the hybrid layer, suggests that the OS ED Pd-NPs/ChOx/Naf-modified electrodes do not operate in an enzyme-limited regime, contrary to the initial assumption.

Moreover, these findings indicate that the sensitivity of OS ED Pd-NPs/ChOx/Naf-modified electrodes may be independent of layer thickness and architecture (see also Section 3.4).

### 3.3 Does DET Mechanism Occur in the Ultrathin Pd-NPs/ChOx/Naf Biosensing Layer?

Since neither the Pd-NPs nor the amount of loaded enzyme (ChOx) significantly affected the performance of OS ED Pd-NPs/ChOx/Naf-modified electrodes (*see previous sections*), it became essential to clarify which factors actually govern their behavior. So far, the thickness of the sensing layer does not exceed micrometer (μm) level, depending on the concentration of the enzyme used in the multiple electrolyte solution; therefore, direct contact between the codeposited enzyme and the electrode cannot be excluded.^13^

To verify the principal possibility of DET within the OS ED hybrid biosensing layer, individual FAD (the active cofactor of ChOx) was electrodeposited onto the electrode together with Pd-NPs and Naf (under the same conditions used for electrodeposition of ChOx), resulting in the formation of the OS ED Pd-NPs/FAD/Naf layer.

The presence of FAD was clearly visualized by a symmetric FADH_2_/FAD redox couple in CV mode, appearing in the cathodic region at – 0.3 V under oxygen-free conditions (OXCAL buffer), as shown in Figure 6A (*plot a*). This is a classic demonstration (electrochemical fingerprint) of DET,^35,36^ without the enzyme.

**Figure 6:**
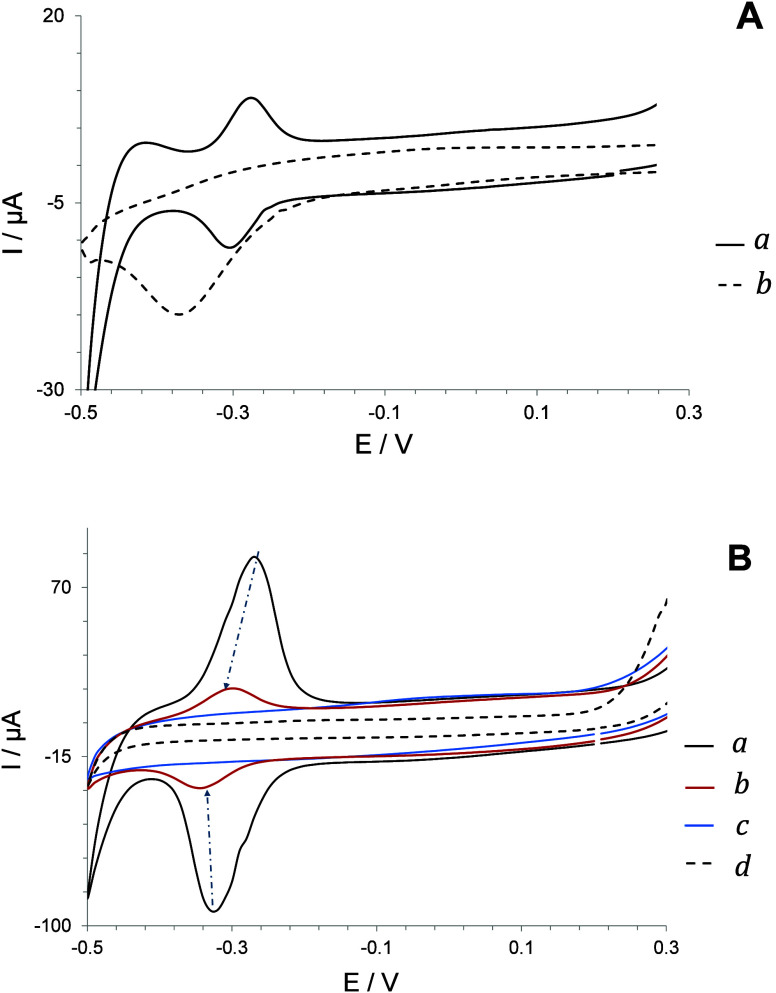
(**A**) CV plots (*2D* *scan shown*) recorded at 20 mV/s from OS ED Pd-NPs/FAD/Naf-modified electrode: (*a*) – in the absence of oxygen (OXCAL buffer) and (*b*) – in the presence of oxygen. (**B**) CV plots (*2D* *scan shown*) recorded at 100 mV/s in the absence of oxygen (OXCAL buffer) from SPEs/GO modified with (*a*) – OS ED Pd-NPs/FAD/Naf; (*b*) – OS ED Pd-NPs/ChOx/FAD/Naf layer; (*c*) – OS ED Pd-NPs/ChOx/Naf layer; (*d*) – OS ED Pd-NPs. *Note*: the pH of all solutions was adjusted to 6.90 ± 0.2.

In contrast, in the presence of oxygen, the ongoing ORR suppressed the visualization of the FAD/FADH_2_ couple (Figure 6A, *plot b*). These results indicate that, for ultrathin films (∼20 nm), dissolved oxygen suppresses the DET pathway involving the FAD/FADH_2_ couple due to competitive oxygen reduction catalyzed by Pd-NPs. In other words, even when the cofactor is positioned in close proximity to the electrode and the sensing layer thickness is minimized, the presence of oxygen kinetically suppresses the FAD/FADH_2_-based DET pathway even in the absence of a protein (enzyme).

Further experiments performed with electrodeposited ChOx (containing its intrinsic FAD cofactor) and the additional exogenous FAD in the same electrolyte, leading to the formation of a Pd-NPs/ChOx/FAD/Naf layer, confirmed a pronounced decrease in DET in the presence of the immobilized enzyme. Accordingly, even under anaerobic conditions (OXCAL buffer), a significant reduction of the characteristic signal at – 0.3 V was observed (Figure 6B).

Moreover, CV plots recorded in OXCAL buffer from the OS ED Pd-NPs/ChOx/Naf-modified electrode in the absence of exogenous FAD showed no evidence of direct FAD/FADH_2_ redox activity, as the characteristic symmetric peak was no longer observed. This behavior is most likely due to masking of the intrinsic FAD/FADH_2_ redox center by the protein matrix (ChOx) since FAD is deeply embedded within the enzyme structure. Consequently, DET to the electrode is effectively hindered despite the ultrathin nature of the biosensing layer and the absence of oxygen.

To sum it up, having excluded diffusion limitations (Figure 2), electrocatalyst-related (Pd-NPs) limitations (Figure 4), enzyme loading limitations (Figure 5), and the absence of a DET mechanism (Figure 6), it can be assumed that the electrode modified with the OS ED Pd-NPs/ChOx/Naf layer operates in an interface-limited regime, where the number of electrochemically accessible Pd–enzyme contacts determines the maximum achievable signal.^37^

### 3.4 Revealing Pd-Enzyme Coupling Interface Limitations

It should be noted that increasing the thickness of the biosensing layer does not necessarily improve the electrochemical signal when the limiting factor is the number of effective enzyme–electrode contacts. Thicker enzyme membranes can impede diffusion of substrate and products to the electrode, resulting in diffusion-controlled current rather than enhanced signal with additional enzyme loading. Moreover, only enzymes that are in direct or mediated electronic communication with the electrode contribute to the measured current, such that increasing the layer thickness without improving contact pathways yields little benefit.^22,38^

To verify this assumption, a layer-by-layer (LbL)-designed electrode was fabricated based on ED Pd-NPs produced at – 2.5 mA for 30 s, followed by drop-coating with 3 μM ChOx and 3 μM Naf. The biosensing layer was dried for 12 h at +4 °C, carefully rinsed with DI water, and then tested with cholesterol solutions under the same conditions used for the electrode modified with the ED Pd-NPs/ChOx/Naf layer. The thickness of the LbL-modified electrode was in the μm range, compared to ∼ 20 nm for the OS ED Pd-NPs/ChOx/Naf layer, resulting in changes in the mass-transfer process within the film (SI, Figure S5A,B; see in comparison with Figure 2A,B).

No linear dependence of the cathodic peak current (I_pc_) on the scan rate in the range of 5–500 mV/s was observed for the LbL design (R^2^ ≈ 0.94) in the presence of oxygen. However, the plot of I_pc_ versus √ν showed an excellent linear correlation (R^2^ > 0.99; SI, Figure S5C), indicating a diffusion-controlled process in this system, in contrast to the adsorption-controlled behavior observed for the OS ED hybrid layer.

Notably, even the use of a multilayer LbL design does not lead to any enhancement in cholesterol sensitivity or LDR (equal to 100 μM – 3 mM, SI, Figure S6), which is comparable to the sensitivity (∼1.0 – 1.2 μA·μM^–1^·cm^–2^) obtained with the OS ED design (see also Figure 4A, Figure 5 and Table S1). This is believed to be caused by a large portion of the enzyme that remains “inactive” from the perspective of the electrochemical signal. In other words, increasing enzyme loading does not improve the analytical signal, as the system operates under an interface-limited H_2_O_2_ utilization regime. Additional enzyme molecules cannot contribute to the signal because the generated H_2_O_2_ cannot reach the active Pd sites within the ultrathin, adsorption-controlled film.

Limitations in Pd–enzyme coupling interface indicate that only a fraction of the immobilized enzyme is functionally connected to the Pd-NPs, regardless of the total enzyme loading. Importantly, this experiment suggests that the observed Pd–enzyme coupling is not specific to the OS ED approach but is also similar for the LbL design, highlighting that Pd–enzyme coupling limitations persist irrespective of the immobilization strategy used.

In other words, regardless of the dominant process in the biosensing layer – adsorption-controlled behavior for the OS ED layer vs diffusion-controlled behavior for the LbL layer, and regardless of the loaded enzyme amount, the cholesterol sensitivity is independent of layer architecture.

To summarize, increasing enzyme loading within the Pd–ChOx interface is not the key factor governing the performance of electrodes modified with the OS ED Pd-NPs/ChOx/Naf layer. Instead, improving the number of functional Pd–ChOx contacts within this ultrathin hybrid biosensing layer appears to be the most promising strategy. For example, replacing the stiff Nafion with a more flexible polymer matrix such as chitosan could enhance Pd–ChOx coupling, a strategy we plan to investigate in future studies to further improve enzyme–electrocatalyst communication and overall sensor performance.

## 4 Conclusions

In this work, a cholesterol biosensor based on a one-step electrodeposited Pd-NPs/ChOx/Naf hybrid ultrathin layer was successfully fabricated and systematically investigated. The developed electrode exhibited a cholesterol sensitivity of approximately 1.0–1.2 μA·μM^–1^·cm^–2^ within a linear detection range of 100 μM to 3 mM. Beyond its analytical merit, this model system served as a platform for elucidating the fundamental processes governing signal formation in hybrid Pd–enzyme interfaces, including direct electron transfer (DET), the oxygen reduction reaction (ORR), and the intrinsic constraints of electrocatalyst–enzyme coupling.

The results reveal that oxygen-related processes dominate the fundamental limitations on DET at Pd–enzyme interfaces. While oxygen interference in oxidase-based electrodes is a known phenomenon, our study provides systematic, quantitative evidence in ultrathin hybrid Pd-NPs/ChOx/Naf films, showing that DET is not established even under optimized nanoscale architectures. These limitations originate from oxygen interference and protein-induced masking of redox-active FAD centers, effects that persist even in ultrathin hybrid films. Importantly, increasing enzyme loading does not proportionally improve sensor performance, since the electrochemical response is determined by the number of functionally coupled Pd–enzyme pairs rather than by the total amount of immobilized enzyme.

Collectively, these findings provide a mechanistic explanation for the nonscaling behavior of the electrochemical signal read-out and emphasize the decisive role of nanoscale Pd–enzyme organization in defining biosensor sensitivity. The fraction of enzyme molecules that are effectively electronically coupled to Pd-NPs, rather than enzyme concentration or layer thickness, ultimately represents the limiting factor governing electrode performance.

## Supplementary Material



## Notes

Oxygen consumption studies in cholesterol-containing droplets; TEM and AFM characterization of Pd-NPs/ChOx/Naf biosensing layers; electrochemical characterization of LbL-designed electrodes; cyclic voltammetry studies at various scan rates; cholesterol calibration plots for LbL- and OS-designed biosensors; analytical figures of merit of the OS-designed Pd-NPs/ChOx/Nafion biosensor; supplementary Figures S1–S6 and Table S1 (PDF)

## Abbreviations

Pd-NPs: palladium nanoparticles
ChOx: cholesterol oxidase
Naf: Nafion
SPEs: screen-printed electrodes
GO: graphene oxide
OS: one step
ED: electrodeposition
LbL: layer-by-layer
TEM: Transmission electron microscopy
AFM: Atomic force microscopy
QCM: quartz microbalance
CV: cyclic voltammetry
AM: amperometry
MAM: multistep amperometry
CP: chronopotentiometry
ORR: oxygen reduction reaction
LDR: linear dynamic range
DET: direct electron transfer
FAD: flavin adenine dinucleotide
ECSA: electroactive surface area

## References

[1] Haritha V.S., Kumar S.R. S., Rakhi R.B. (2022). Amperometric Cholesterol Biosensor
Based on Cholesterol Oxidase and Pt-Au/MWNTs Modified Glassy Carbon
Electrode. Mater. Today Proc..

[2] Cevik E., Cerit A., Gazel N., Yildiz H. B. (2018). Construction of
an Amperometric Cholesterol Biosensor Based on DTP­(Aryl)­Aniline Conducting
Polymer Bound Cholesterol Oxidase. Electroanalysis.

[3] Ghosh S., Ahmad R., Khare S. K. (2018). Immobilization of Cholesterol Oxidase:
An Overview. Open Biotechnol. J..

[4] Vidal J.-C., Espuelas J., Garcia-Ruiz E., Castillo J.-R. (2004). Amperometric Cholesterol
Biosensors Based on the Electropolymerization of Pyrrole and the Electrocatalytic
Effect of Prussian-Blue Layers Helped with Self-Assembled Monolayers. Talanta.

[5] Saxena U., Chakraborty M., Goswami P. (2011). Covalent Immobilization
of Cholesterol
Oxidase on Self-Assembled Gold Nanoparticles for Highly Sensitive
Amperometric Detection of Cholesterol in Real Samples. Biosens. Bioelectron..

[6] Trujillo R. M., Barraza D. E., Zamora M. L., Cattani-scholz A., Madrid R. E. (2021). Nanostructures in Hydrogen Peroxide
Sensing. Sensors.

[7] Chen A., Chatterjee S. (2013). Nanomaterials Based Electrochemical Sensors for Biomedical
Applications. Chem. Soc. Rev..

[8] Chen W., Cai S., Ren Q.-Q., Wen W., Zhao Y.-D. (2012). Recent Advances
in Electrochemical Sensing for Hydrogen Peroxide: A Review. Analyst.

[9] Pollegioni L., Gadda G., Ambrosius D., Ghisla S., Pilone M. S. (1999). Cholesterol
Oxidase from Streptomyces Hygroscopicus and Brevibacterium Sterolicum:
Effect of Surfactants and Organic Solvents on Activity. Biotechnol. Appl. Biochem..

[10] Sharma D., Lee J., Seo J., Shin H. (2017). Development of a Sensitive Electrochemical
Enzymatic Reaction-Based Cholesterol Biosensor Using Nano-Sized Carbon
Interdigitated Electrodes Decorated with Gold Nanoparticles. Sensors (Basel)..

[11] Arya S. K., Datta M., Malhotra B. D. (2008). Recent Advances in Cholesterol Biosensor. Biosens. Bioelectron..

[12] Silina Y. E., Apushkinskaya N., Talagaeva N. V., Levchenko M. G., Zolotukhina E. V. (2021). Electrochemical
Operational Principles and Analytical
Performance of Pd-Based Amperometric Nanobiosensors. Analyst.

[13] Koch M., Korkmaz N., Silina Y. E. (2025). Investigation
of the Binding Kinetics
and Electrochemical Properties of in Situ Reconstructed Apo-GOx Using
Electrodes with Electrodeposited. Analyst.

[14] Schachinger F., Chang H., Scheiblbrandner S., Ludwig R. (2021). Amperometric Biosensors
Based on Direct Electron Transfer Enzymes. Molecules..

[15] Haghighi B., Tabrizi M. A. (2013). Direct Electron
Transfer from Glucose Oxidase Immobilized
on an Overoxidized Polypyrrole Film Decorated with Au Nanoparticles. Colloids Surfaces B Biointerfaces.

[16] Malekzad H., Sahandi Zangabad P., Mirshekari H., Karimi M., Hamblin M. R. (2017). Noble Metal
Nanoparticles in Biosensors: Recent Studies and Applications. Nanotechnology Rev..

[17] Tavahodi M., Ortiz R., Schulz C., Ekhtiari A., Ludwig R., Haghighi B., Gorton L. (2017). Direct Electron
Transfer of Cellobiose
Dehydrogenase on Positively Charged Polyethyleneimine Gold Nanoparticles. ChemPlusChem..

[18] Silina Y. E. (2024). One-Step
Electrodeposited Hybrid Nanofilms in Amperometric Biosensor Development. Anal. Methods.

[19] Silina Y. E., Apushkinskaya N., Talagaeva N. V., Levchenko M. G., Zolotukhina E. V. (2021). Electrochemical
Operational Principles and Analytical
Performance of Pd-Based Amperometric Nanobiosensors. Analyst.

[20] Silina Y. E., Butyrskaya E. V., Koch M., Fink-Straube C., Korkmaz N., Levchenko M. G., Zolotukhina E. V. (2024). Mechanistic
Aspects of Glycerol Oxidation on Palladium Electrocatalysts in Model
Aqueous and Fermentation Media Solutions. Electrochim.
Acta.

[21] Zolotukhina E. V., Butyrskaya E. V., Koch M., Herbeck-Engel P., Levchenko M. G., Silina Y. E. (2023). First Principles of Hydrazine Electrooxidation
at Oxide-Free and Oxide-Based Palladium Electrodes in Complex Media. Phys. Chem. Chem. Phys..

[22] Baronas R., Ivanauskas F., Kulys J. (2003). The Influence of the Enzyme Membrane
Thickness on the Response of Amperometric Biosensors. Sensors.

[23] Dewangan L., Korram J., Karbhal I., Nagwanshi R., Jena V. K., Satnami M. L. (2019). A Colorimetric Nanoprobe Based on
Enzyme-Immobilized Silver Nanoparticles for the Efficient Detection
of Cholesterol. RSC Adv..

[24] El-Naggar N. E.-A., Deraz S. F., Soliman H. M., El-Deeb N. M., El-Shweihy N. M. (2017). Purification,
Characterization and Amino Acid Content of Cholesterol Oxidase Produced
by Streptomyces Aegyptia NEAE 102. BMC Microbiol..

[25] Bokoch M. P., Devadoss A., Palencsár M. S., Burgess J. D. (2004). Steady-State Oxidation
of Cholesterol Catalyzed by Cholesterol Oxidase in Lipid Bilayer Membranes
on Platinum Electrodes. Anal. Chim. Acta.

[26] Hu J., Huang X., Xue S., Yesilbas G., Knoll A., Schneider O. (2020). Measurement
of the Mass Sensitivity of QCM with Ring
Electrodes Using Electrodeposition. Electrochem.
commun..

[27] Fu K., Seo J.-W., Kesler V., Maganzini N., Wilson B. D., Eisenstein M., Murmann B., Soh H. T. (2021). Accelerated
Electron Transfer in Nanostructured Electrodes Improves the Sensitivity
of Electrochemical Biosensors. Adv. Sci. (Weinheim,
Baden-Wurttemberg, Ger..

[28] Chen H., Simoska O., Lim K., Grattieri M., Yuan M., Dong F., Lee Y. S., Beaver K., Weliwatte S., Gaffney E. M., Minteer S. D. (2020). Fundamentals,
Applications,
and Future Directions of Bioelectrocatalysis. Chem. Rev..

[29] Yan X., Tang J., Tanner D., Ulstrup J., Xiao X. (2020). Direct Electrochemical
Enzyme Electron Transfer on Electrodes Modified by Self-Assembled
Molecular Monolayers. Catalysts.

[30] Cui H.-F., Zhang K., Zhang Y.-F., Sun Y.-L., Wang J., Zhang W.-D., Luong J. H. T. (2013). Immobilization
of Glucose Oxidase
into a Nanoporous TiO2 Film Layered on Metallophthalocyanine Modified
Vertically-Aligned Carbon Nanotubes for Efficient Direct Electron
Transfer. Biosens. Bioelectron..

[31] Wang J. (2006). Electrochemical
Biosensors: Towards Point-of-Care Cancer Diagnostics. Biosens. Bioelectron..

[32] Molaei R., Sabzi R. E., Farhadi K., Kheiri F., Forough M. (2014). Amperometric
Biosensor for Cholesterol Based on Novel Nanocomposite Array Gold
Nanoparticles/Acetone-Extracted Propolis/Multiwall Carbon Nanotubes/Gold. Micro Nano Lett..

[33] Dey R. S., Raj C. R. (2010). Development of an
Amperometric Cholesterol Biosensor
Based on Graphene-Pt Nanoparticle Hybrid Material. J. Phys. Chem. C.

[34] Umar A., Ahmad R., Hwang S. W., Kim S. H., Al-Hajry A., Hahn Y. B. (2014). Development of Highly
Sensitive and Selective Cholesterol
Biosensor Based on Cholesterol Oxidase Co-Immobilized with α-Fe2O3Micro-Pine
Shaped Hierarchical Structures. Electrochim.
Acta.

[35] Bollella P. (2020). Enzyme-Based Biosensors: Tackling Electron Transfer Issues. Sensors.

[36] Yin Y., Lü Y., Wu P., Cai C. (2005). Direct Electrochemistry
of Redox Proteins and Enzymes Promoted by Carbon Nanotubes. Sensors.

[37] Cao X., Ye Y., Li Y., Xu X., Yu J., Liu S. (2013). Self-Assembled
Glucose Oxidase/Graphene/Gold Ternary Nanocomposites for Direct Electrochemistry
and Electrocatalysis. J. Electroanal. Chem..

[38] Saboe P. O., Conte E., Farell M., Bazan G. C., Kumar M. (2017). Biomimetic
and Bioinspired Approaches for Wiring Enzymes to Electrode Interfaces. Energy Environ. Sci..

