# Supplementary material for: Interface-Limited Amperometric Cholesterol Biosensing in Ultrathin Pd-NPs-Based-Enzyme Films
Source: Langmuir. 2026 Jul 10;42(29):21039–48. doi: 10.1021/acs.langmuir.6c01437 (PMC13421975; doi:10.1021/acs.langmuir.6c01437)
Supplement: Supplementary file 1 [file la6c01437_si_001.pdf]

Supporting Information

Interface-Limited Amperometric Cholesterol Biosensing in Ultrathin Pd-NPs-based-Enzyme Films

Y.E. Silina[1], M. Koch[2], N. Korkmaz[3]*

[1]Saarland University, Department of Biochemistry, Campus B2.2, 66123, Saarbrücken, Germany
[2]HTW saar – University of Applied Sciences, 66117, Saarbrücken, Germany
[3]KIST Europe – AI Convergence Cluster, Korea Institute of Science and Technology, 66123, Saarbrücken, Germany

**The correspondence*
should be addressed (Y.E. Silina)
Institute of Biochemistry,
Saarland University, Saarbrücken, Germany
Campus B 2.2, room 317
E-mail 1: yuliya.silina@gmx.de
E-mail 2: yuliya.silina@uni-saarland.de

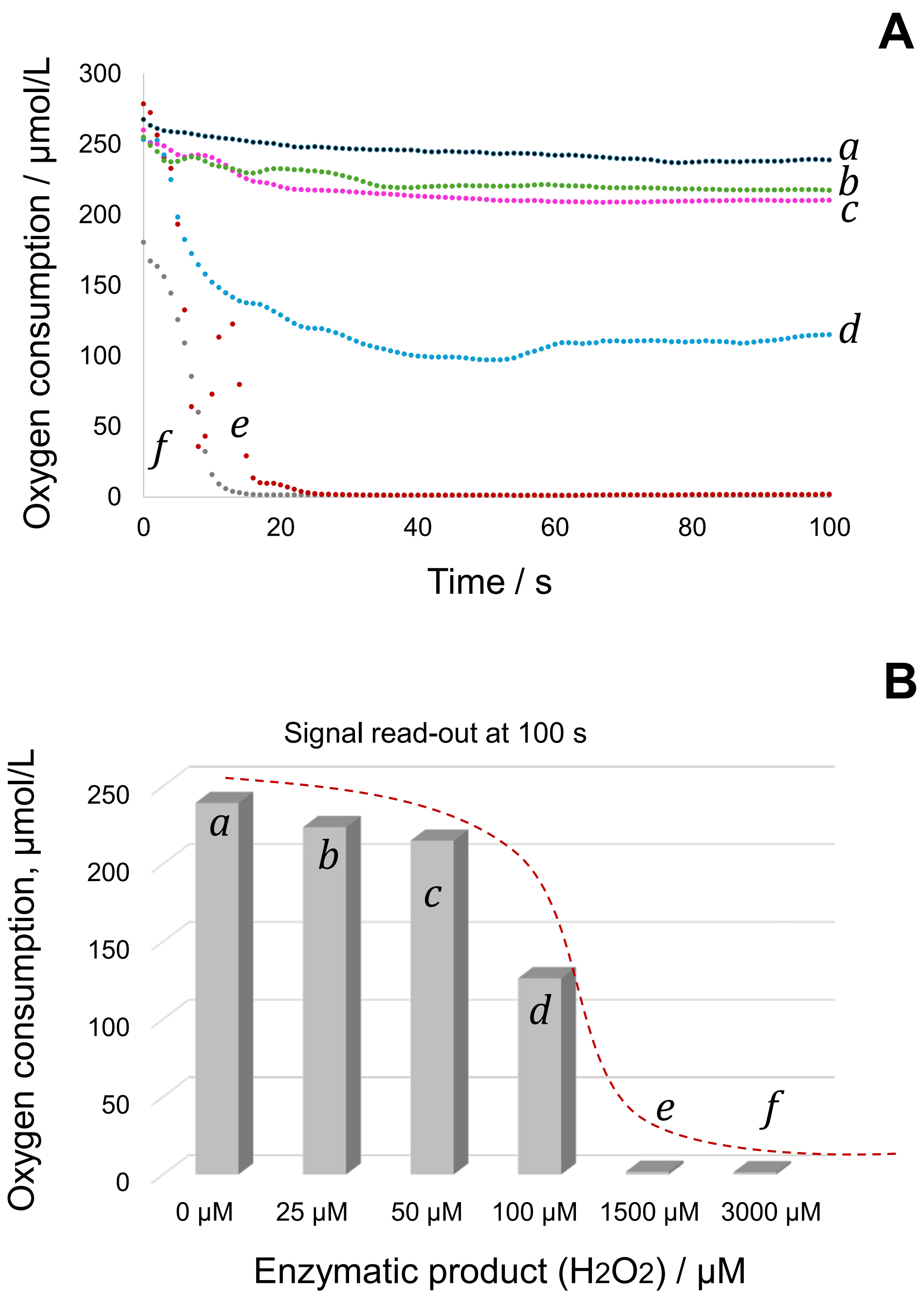

Fig. S1 – (**A**) Oxygen consumption (µmol/L) at 20 ± 2 °C in 150 µL cholesterol droplets, without (a) and with aqueous ChOx (b–f): a – 3 mM cholesterol; b – 25 µM + 10 µL ChOx; c – 50 µM + 10 µL ChOx; d – 100 µM + 10 µL ChOx; e – 1.5 mM + 10 µL ChOx; f – 3 mM + 10 µL ChOx. Cholesterol was in a surfactant-rich environment. **(B)** Signal read-out was recorded at 100 s after the addition of 10 µL of aqueous ChOx to cholesterol droplets.

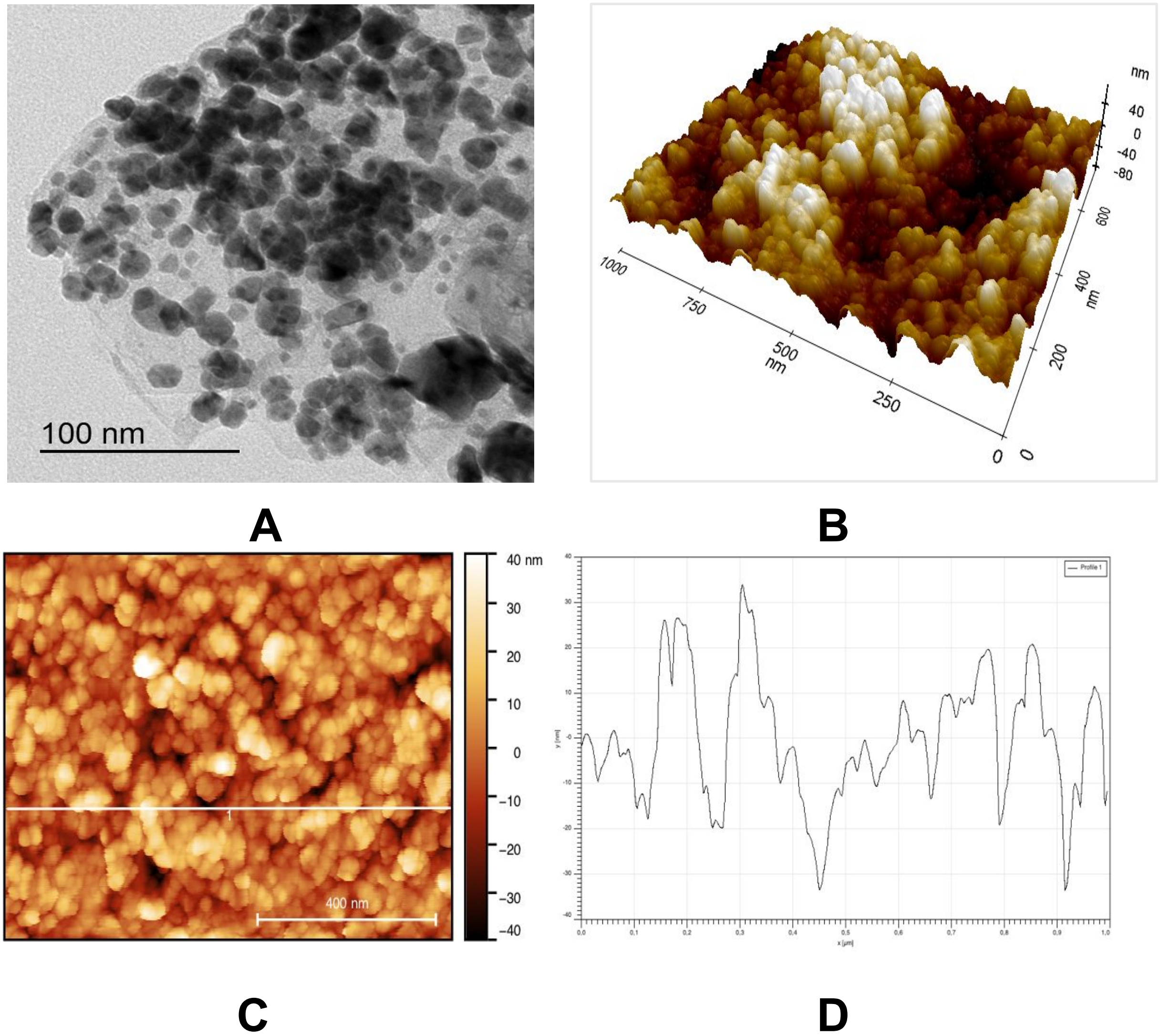

Fig. S2 – TEM (**A**) and AFM (**B,C,D**) images of Pd-NPs/ChOx/Naf biosensing layer deposited on SPEs/GO. *Note:* an average layer thickness for the electrodeposited layer was estimated to be 18.4±9.7 nm.

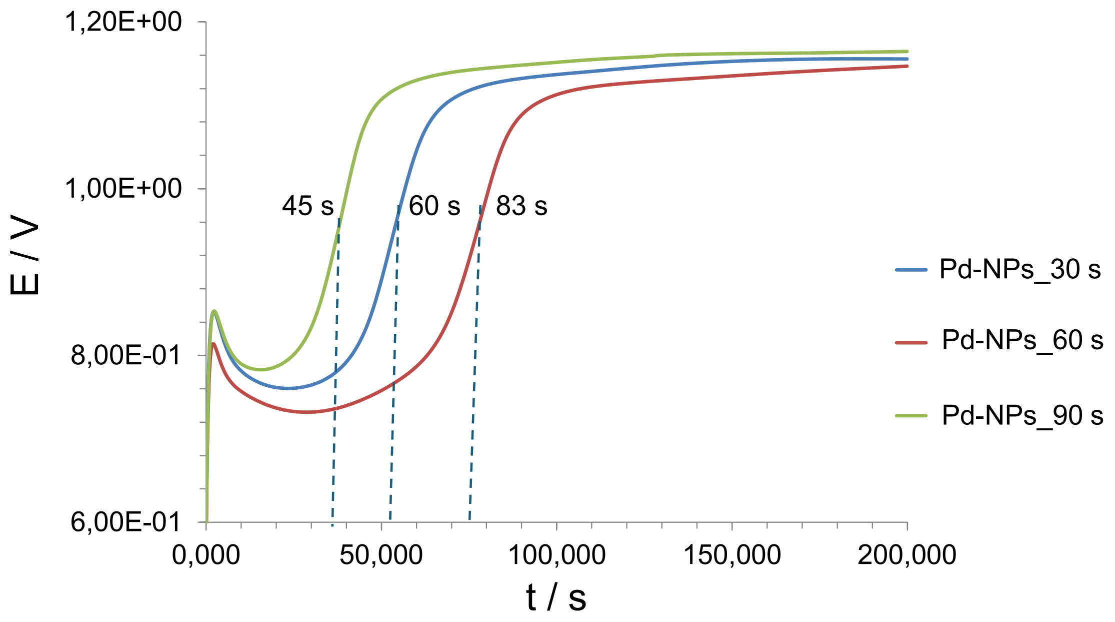

Fig. S3 – CP curves recorded in chronopotentiometric mode in 1 M HCl at 0.1 mA for electrodes modified with Pd-NPs deposited onto SPEs/GO at −2.5 mA for 30, 60, and 90 s. The dissolution times of Pd-NPs deposited at −2.5 mA for 30, 60, and 90 s were 45 s, 60 s, and 83 s, respectively. The deposited Pd masses were calculated from these CP curves using Faraday's law and were 1.93, 3.03, and 4.14 µg, respectively.

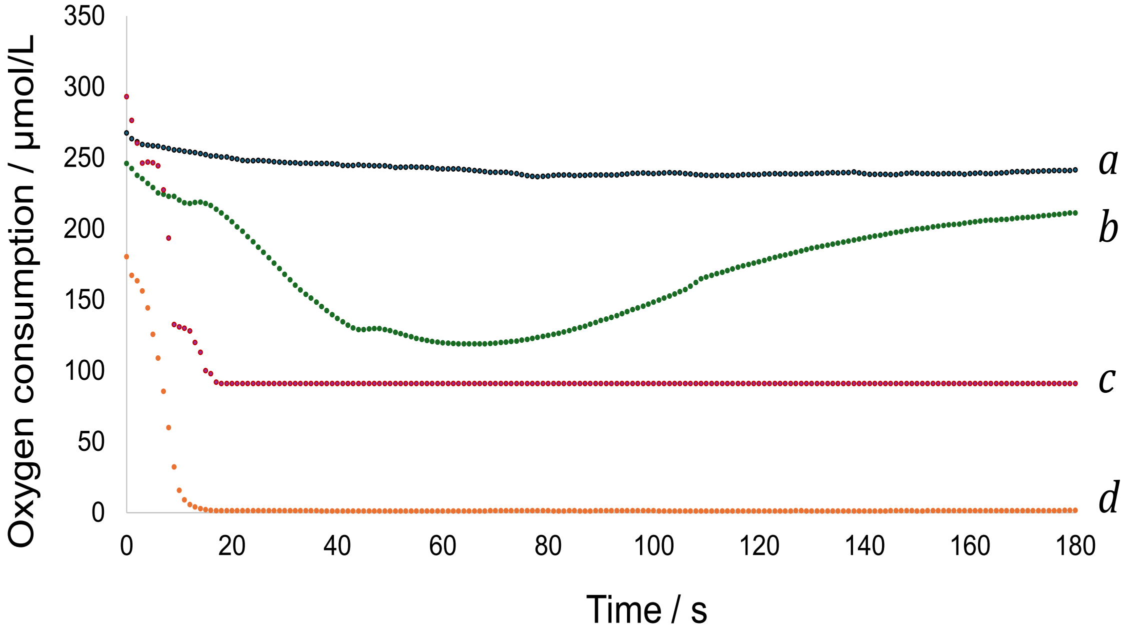

Fig. S4 – Oxygen consumption (µmol/L) at 20±2 °C in 150 µL cholesterol droplets, without ChOx (*a*) and with added multiple electrolytes containing aqueous ChOx (10 KU) (*b,c,d*)at various concentrations: *b* – 2 mM + 10 µL mix containing ChOx (1 mg/mL), *c* – 2 mM + 10 µL mix containing ChOx (4 mg/mL); *d* – 2 mM + 10 µL mix of individual ChOx (4 mg/mL). *Note:* concentration of Pd-electrolyte and Nafion in (*b*) and (*c*) was kept at the constant level in all cases.

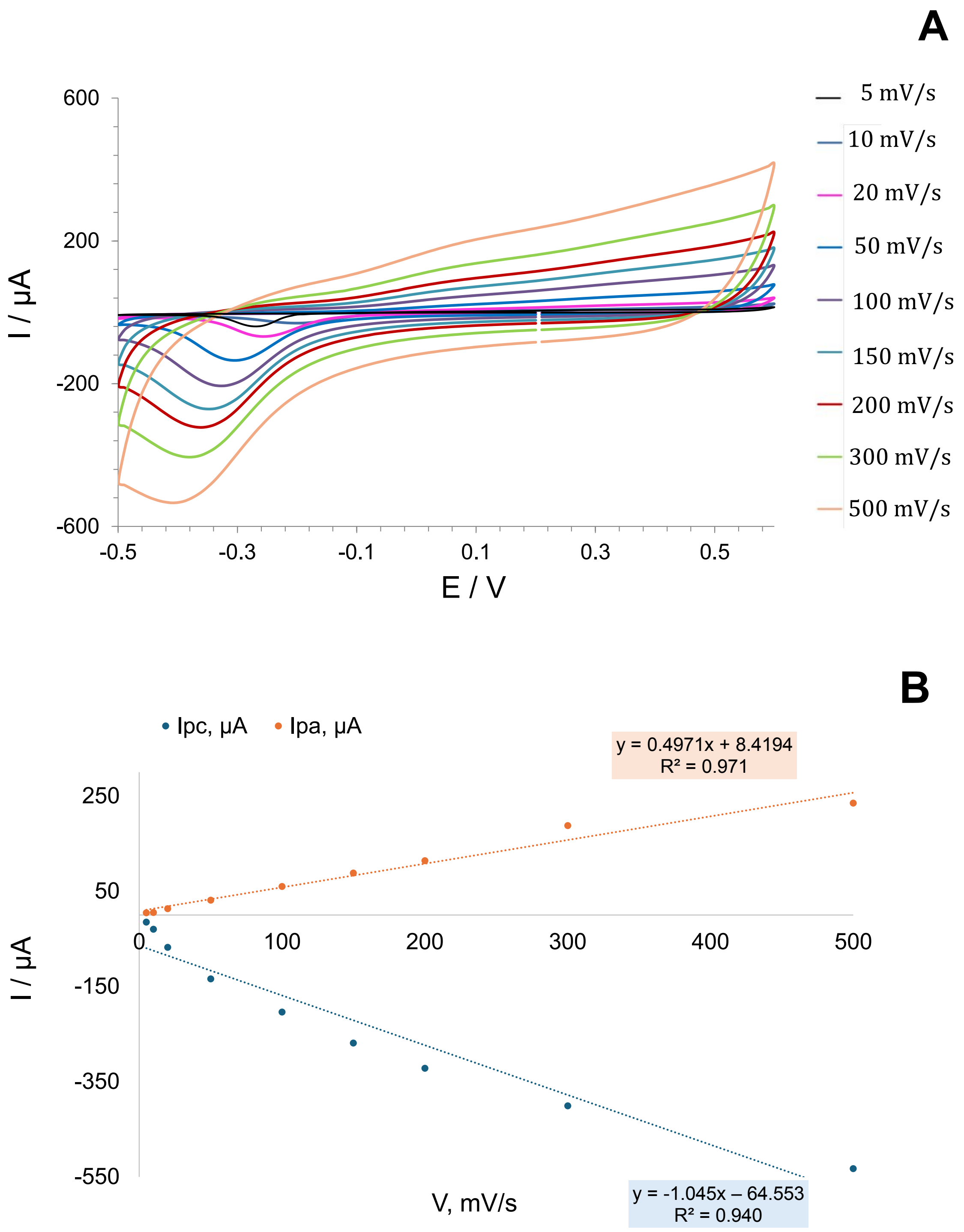
A
600
200
-200
-600
I / µA
-0.5
-0.3
-0.1
0.1
0.3
0.5
E / V
5 mV/s
10 mV/s
20 mV/s
50 mV/s
100 mV/s
150 mV/s
200 mV/s
300 mV/s
500 mV/s
B
Ipc, µA
Ipa, µA
y = 0.4971x + 8.4194
R² = 0.971
250
50
-150
-350
-550
I / µA
0
100
200
300
400
500
V, mV/s
y = -1.045x – 64.553
R² = 0.940

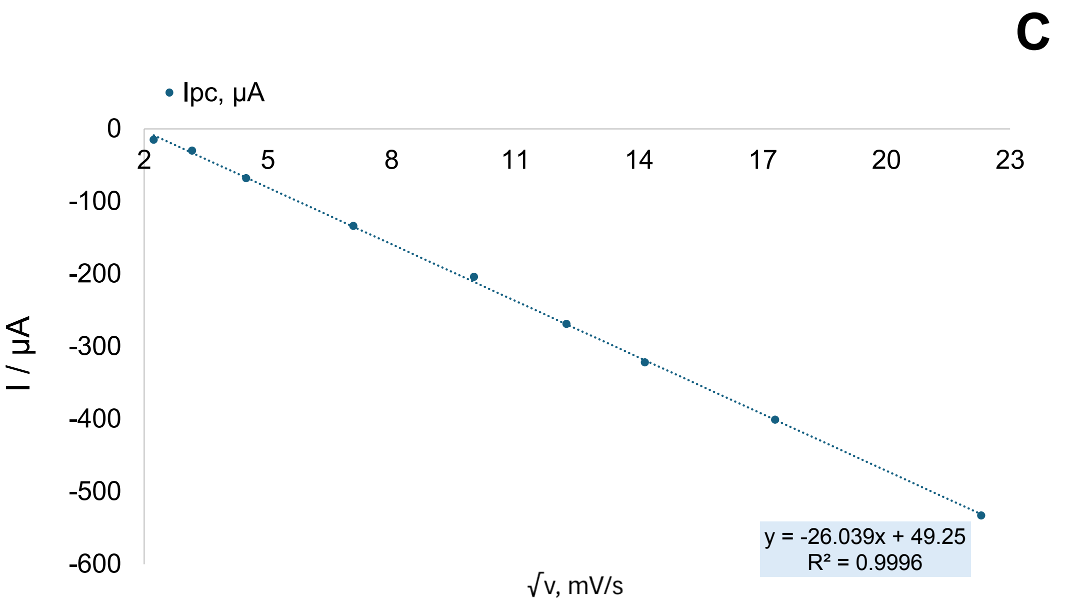

Fig. S5 – (**A**) Cyclic voltammograms (2d scans shown) obtained from LbL Pd-NPs/ChOx/Naf-modified electrode in phosphate buffer (pH 7.0±0.2) at various scan rates measured from 5 to 300 mV s^{-1} (from inner to outer profiles), respectively, and (**B**) plots of peak currents *vs*. the scan rates (*vs*. Ag/AgCl) at the ambient conditions (**C**) plots of peak currents *vs*. √v, mV/s (Ag/AgCl) at the ambient conditions.

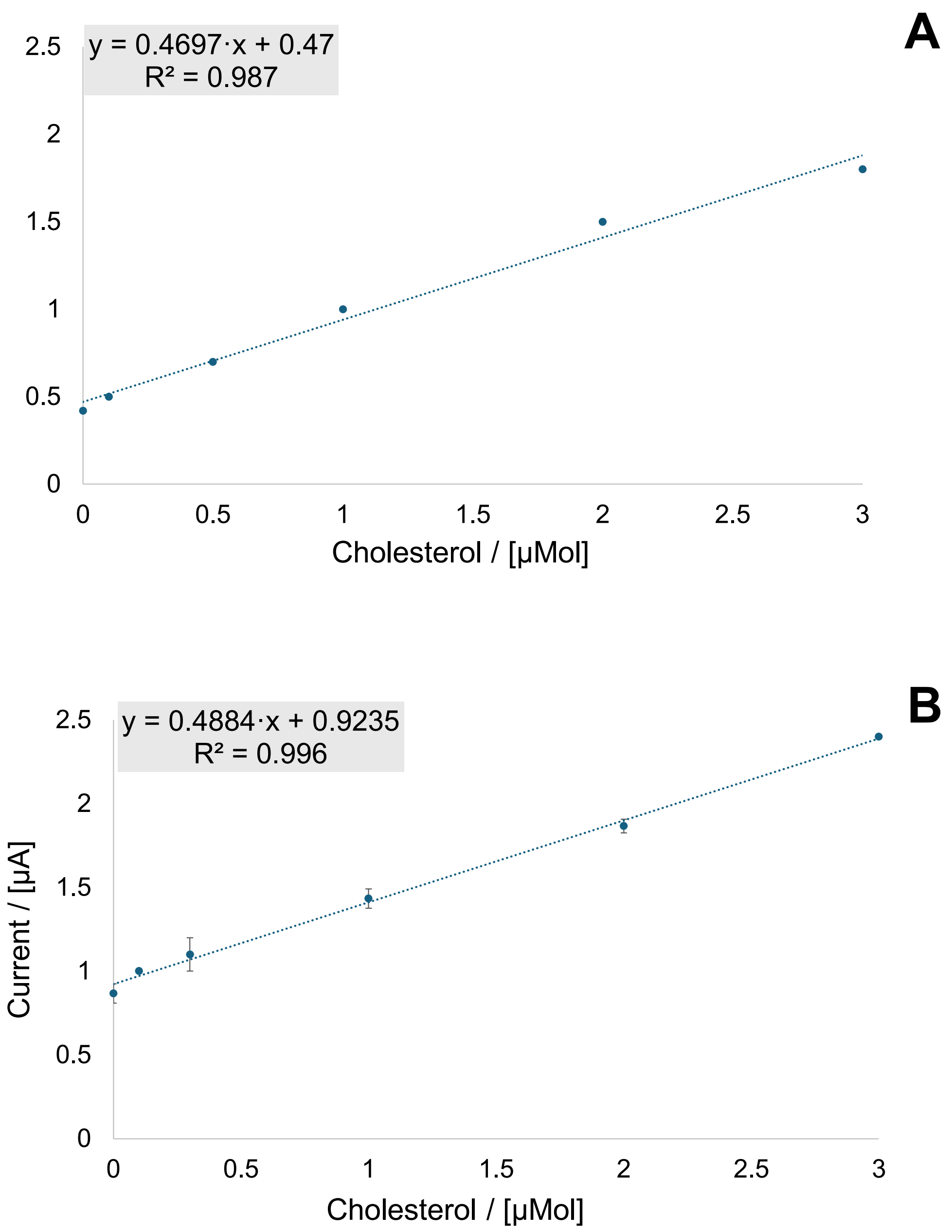

Fig. S6 – Cholesterol calibration plots (with calibrants prepared in mixture A) were recorded from the following electrodes: **A** – LbL-designed electrode based on drop-coated ChOx and electrodeposited Pd-NPs-modified SPE; **B** – OS designed Pd-NPs/ChOx/Naf-modified SPE.

Table S1. Selected analytical figures of merit of the OS-designed Pd-NPs/ChOx/Nafion biosensor evaluated in a cholesterol-containing *mixture A* as the matrix

Sensitivity	Linear dynamic range	Limit of quantification	Calibration formula	Regression coefficient, R^2
1.0 – 1.2 $\mu A \cdot \mu M^{-1} \cdot cm^{-2}$	100 µM – 3 mM	80±5 µM	$y = 0.4884 \cdot x + 0.9235$	0.996
